# Consider or not consider: the unsolved question on the use of radioactive iodine for differentiated thyroid cancer with low to intermediate risk of recurrence

**DOI:** 10.1007/s12020-024-04027-4

**Published:** 2024-09-26

**Authors:** Pasqualino Malandrino, Dario Tumino, Marco Russo, Rosario Le Moli, Antonio Prinzi, Tommaso Piticchio, Francesco Frasca

**Affiliations:** 1 Endocrinology Unit, Department of Clinical and Experimental Medicine, University of Catania, Garibaldi-Nesima Medical Hospital, Catania, Italy; 2 Department of Medicine and Surgery, University of Enna “Kore”, Enna, Italy

**Keywords:** differentiated thyroid carcinoma, radioactive iodine, recurrent disease, predictive factors, treatment effect

## Abstract

**Background:**

Surgery stands as the cornerstone treatment for differentiated thyroid cancer (DTC). After surgery, radioactive iodine (RAI) administration is primarily recommended for high-risk patients and commonly employed to address residual disease or mitigate the risk of recurrence. However, the optimal application of RAI in cases categorized as low to intermediate risk is still uncertain. This study aims to assess the indication of post-surgical RAI treatment specifically in patients diagnosed with DTC falling within the low to intermediate risk category for recurrent disease.

**Methods:**

retrospective analysis of consecutive patients with DTC falling within the low to intermediate risk category for recurrence and diagnosed between 2009–2015. Patients were categorized into either treated or untreated with RAI. Treatment effect was assessed by the inverse-probability weighted regression adjustment (IPWRA), by balancing the distribution of factors influencing outcome and treatment assignment.

**Results:**

after surgery, 328 patients (69.9%) were treated with RAI while 141 (30.1%) were left untreated. Across the entire cohort, 44 individuals (9.4%) displayed biochemical or structural disease after a median time of 17.5 months following diagnosis. Recurrent disease was more prevalent in patients who underwent RAI treatment compared to those untreated (12.5% vs 2.1%, respectively, *p* < 0.001). Factors independently associated with recurrent disease, identified through multivariate logistic regression analysis, included lymph node metastases (pN1) (OR = 4.07; 95% CI 1.84–8.97), male sex (OR = 2.71; 95% CI 1.31–5.59), tumor size (OR = 1.03; 95% CI 1.00–1.06), and microscopic extrathyroidal extension (OR = 2.36; 95% CI 1.15–4.81). IPWRA analysis revealed that the occurrence of recurrent disease was 9.6% (95% CI = 6.3–12.9) in RAI-treated patients and 15.9% (95% CI = 11.1–20.71) in untreated patients (*p* = 0.021). As a consequence, if all patients underwent RAI treatment, the estimated risk of recurrence would be reduced by 42% (RR = 0.58; 95% CI = 0.35–0.91, *p* = 0.018). The greatest benefit was observed in patients with 2 intermediate risk factors.

**Conclusions:**

These results suggest that treatment with RAI in low to intermediate DTC can reduce the risk of recurrence in selected patients. However, definitive answers regarding whether to consider RAI therapy for this category of patients can only be attained through prospective clinical trials. Up to date these results recommend a meticulous assessment of tumor characteristics at diagnosis to guide the decision regarding RAI administration.

## Introduction

Differentiated thyroid cancer (DTC) refers to thyroid tumors derived from follicular cells, including papillary thyroid carcinoma (PTC), follicular thyroid carcinoma (FTC), oncocytic carcinoma (OCA), differentiated high-grade thyroid carcinoma (DHGTC) and poorly differentiated thyroid carcinoma (PDTC) [1]. It is the 10th most common cancer with an age-standardized incidence rates of 10.1 per 100,000 in women and 3.1 per 100,000 in men [2–4]. DTC has an estimated 5-year survival of 99.9% for localized disease and 74.2% in patients with distant metastases [5]. There is universal agreement that surgery is the primary treatment, while the extent of surgery is still debated [6]. After surgery, the need for the administration of radioactive iodine (RAI) is evaluated with the purpose of treating persistent disease or ablating normal thyroid tissue remnant. Moreover, RAI may be administered with the aim of reducing the risk of recurrence in patients with no evidence of disease after surgery (i.e. adjuvant therapy). Indeed, as suggested by the American Thyroid Association (ATA) guidelines, each patient is evaluated to assign a risk category according to the tumor characteristics at diagnosis. Two prospective trials indicate that RAI therapy does not reduce the risk of recurrence in patients with low-risk DTC [7, 8], whereas indication for a real benefit of RAI in reducing the events of recurrence or death is documented only in patients at high risk (i.e. patients with gross extrathyroid extension, incomplete tumor resection, distant metastases, post-operative thyroglobulin suggestive of distant metastases, lymph node metastases >3 cm or FTC with extensive vascular invasion) [9]. There is not strong data to support the use of RAI therapy in patients with low to intermediate risk of recurrence, namely patients that have at least one of the following features: tumor size >4 cm, microscopic extrathyroid extension (ETE), N1 with all involved lymph nodes <3 cm in the largest dimension, aggressive histological variant, vascular invasion. According to these pathological features, these patients have an estimated rate of recurrence ranging between 3% and 30% [9] and the current literature, all retrospective studies, are not consistent to demonstrate a real benefit from the RAI therapy [10–12]. Indeed, ATA guidelines uses the term “consider”, inviting physicians to choose whether to treat or not, based on the association of more pathological features related to unfavorable outcomes and advanced age. This lack of guidance of the scientific societies may lead to discrepancies in treatment protocols, with the risk of an over- or under-treatment in a large group of patients who instead need a better tailoring of care strategy. Aims of our study are: 1) To verify whether RAI therapy is effective in reducing the risk of recurrence in patients with indication to consider RAI (low to intermediate risk DTC); 2) To identify factors that can help select patients with low to intermediate risk of recurrence who could potentially benefit from RAI.

## Materials and methods

We retrospectively reviewed our digital medical records of all consecutive patients that referred to our out-patient Thyroid Clinic (Endocrinology, Garibaldi Nesima Medical Center, University of Catania Medical School, Italy) after the diagnosis of DTC (years 2009 to 2015). For study purposes we selected only patients undergone total thyroidectomy with or without neck lymph node dissection with at least three years of follow-up and who met the criteria of the ATA for being classified at low to intermediate risk of recurrence (Table 1), with an estimated risk of recurrent disease of 30% or less at baseline. In detail we included all patients with at least one of the following characteristics after surgery: tumor size more than 4 cm, tumors more than 1 cm with either microscopic ETE or more than 5 metastatic lymph nodes of the neck central compartment or metastatic lymph nodes of the lateral or mediastinal compartments or aggressive histological variant (i.e., tall cell, insular, diffuse sclerosing), vascular invasion or moderately/poorly differentiated thyroid cancer. We excluded the patients with the following features: microcarcinoma (i.e. tumor size ≤ 1 cm), pN1 with largest nodal dimension >3 cm, gross ETE and patients with less than three years of follow-up. Tumor grade was classified according to WHO 4th edition [13]. All patients included in the analysis were disease-free after surgery, based on the following criteria within 12 months post-surgery: undetectable levels of thyroglobulin (Tg) and absence of anti-thyroglobulin antibodies (Tg-Ab) while on levothyroxine therapy, no abnormal findings on neck ultrasound (US), and no abnormal RAI uptake on post-treatment whole-body scan (if performed). To evaluate the indication for the RAI therapy all patients were evaluated by the attending physicians and the final decision was made not only on the basis of the pathological findings but also taking into consideration the following parameters: post-operative Tg and Tg-Ab values on levo-thyroxine therapy, neck US, presence of comorbidities, pregnancy planning, patient’s refusal. Patients treated with RAI received 30 mCi after recombinant human TSH (rhTSH) stimulation or 100 mCi after either rhTSH or levo-thyroxine withdrawal. During the study period, patients were usually evaluated 3 months after surgery and at least once a year thereafter with serum Tg and Tg-Ab assays and a by neck US performed by clinicians with specific experience and training in neck US. Tg and Tg-Ab were measured using commercial kits: serum Tg with a second-generation chemiluminescent Tg immunoassay (Tg Access; Beckman Coulter, Brea, CA, USA) with a functional sensitivity of 0.1 ng/ml and Tg-Ab were measured by an immune-enzymatic assay (Abbott AxSYM anti-Tg). Outcomes at each visit were then classified in terms of response to the initial therapy as excellent (ER), indeterminate (IndR), biochemical incomplete (BIR), and structural incomplete (SIR), as defined in the 2015 ATA guidelines for patients undergoing total thyroidectomy followed by RAI remnant ablation [9] and as advocated by the European Society for Medical Oncology for those whose initial treatment consisted of total thyroidectomy alone or lobectomy [14]. Classification was based on data available immediately after the initial treatment, including results of post RAI whole-body scan, if performed. Cervical lymph nodes were classified as suspicious (presence of microcalcifications, cystic areas, thyroid tissue-like appearance, peripheral vascularization); indeterminate (loss of fatty hilum plus one or more of the following: round shape, increased short axis, or increased central vascularization); or normal (none of the aforementioned features), according to the classification proposed in 2013 by the European Thyroid Association [15]. When at least one lymph node was classified as suspicious, it was evaluated by Tg measurement in the washout fluid from the fine-needle aspiration cytology and considered as evidence of structural disease in case of confirmatory findings. Other imaging studies (i.e. computed tomography, magnetic resonance imaging, positron emission tomography with18-FDG) were performed in case of BIR or SIR.

**Table 1:** Criteria used to select cohort members with an estimated risk of recurrence of 30% or less

	Estimated risk of recurrence		
Baseline findings	Low (< 5%)	Lower-Intermediate (5–10%)	Higher-Intermediate (10–30%)
Intrathyroidal, papillary microcarcinoma, multifocal	3–5%		
Clinical N0 or ≤5 pathologic N1 micrometastases (<0.2 cm in largest dimension)	5%		
Tumor size >4 cm (T3a) limited to the thyroid		3–8%	
Microscopic invasion of tumor into the perithyroidal soft tissues		3–8%	
Aggressive histology (tall-cell, hobnail-cell, columnar-cell, diffuse- sclerosing or solid/trabecular PTC variant)			10–30%
Papillary thyroid cancer with vascular invasion			15–30%
Clinical N1 or >5 pathologic N1 with all involved lymph nodes <3 cm in largest dimension			20%

### Data analysis

The data recorded for each patient were: age, gender, tumor pathology, surgical and RAI treatment, and periodic follow-up examination results. Patients were divided in treated or untreated with RAI.

This research was carried out following the guidelines outlined in the Helsinki Declaration. Approval for the study protocol was obtained from the Institutional Review Board of the Comitato Etico Catania 2. Patients provided informed consent for the utilization of their data in this study.

### Statistical analysis

We summarized the distribution of continuous variables using means and standard deviation or medians with 25–75 interquartile ranges (IQR) for not-normally distributed variables, while nominal variables were described in terms of frequency counts and corresponding percentages. The t-test was used to compare continuous variables and percentages were compared using the χ2 tests. We carried out uni- and multivariate logistic regression analysis to identify the independent factors predictive of biochemical/structural recurrent disease. Independent predictors were identified by a *p*-value < 0.05. Additionally, to minimize the effects of selection bias from non-randomized treatment assignment, the potential outcome mean (POM), average treatment effect (ATE) and odds ratio (OR) were calculated by combining the outcome and treatment models through an inverse probability weight regression adjustment (IPWRA) analysis. The outcome was modeled as a function of sex, tumor size, microscopic ETE and lymph node metastases. The treatment was modeled as a function of grading, multifocality, vascular invasion, aggressive histological variant, microscopic ETE and lymph node metastases. Thereafter, we assessed the standardized differences and considered a value < 10% to be indicative of negligible imbalance in the baseline covariates between treated and untreated subjects [16]. Statistical analyses were carried out using the STATA 18.0 statistical package (StataCorp LP, College Station, Texas, USA).

## Results

### Patient selection and clinical PTC features

We selected 469 patients followed in our institution. Table 2 summarizes their clinicopathological features at diagnosis. Most of them were female (74.6%) and the mean age at diagnosis was 46.1 ± 14.3 years old. The papillary histotype was predominant (96.8%). Median post-operative Tg was 0.3 (IQR 0.1–0.7). Features of more aggressive biological behavior were identified as follows: aggressive histological variant in 7.9% of patients, moderately or poorly differentiated grade in 10%, positive margin of surgical resection (i.e., R1) in 3.2%, tumor size larger than 4 cm in 3.5%, multifocal tumor in 29.9%, vascular invasion in 9.6%, microscopic ETE in 32.6%, lymph node metastasis in 38.2%. Among the total of 469 PTC patients, 328 were treated with radioiodine (RAI Group) and 141 were untreated (No RAI Group) (Table 3). Out of 328 patients who underwent RAI treatment, 52 (15.9%) received a dosage of 30 mCi, while 276 (84.1%) received a dosage of 100 mCi. At diagnosis no differences were observed between the two groups in terms of male to female ratio, age, histotype, tumor size and follow-up length (Table 3). As expected, patients of the RAI Group had more frequently than patients of the No RAI Group some worrisome features such as: aggressive histological variant (10.1% vs 2.8%, respectively; *p* = 0.008), advanced tumor grade (12.8% vs 3.5%, respectively; *p* = 0.023), multifocality (36.0% vs 15.6%, respectively; *p* < 0.001), vascular invasion (12.2% vs 3.5%, respectively; *p* = 0.004), microscopic ETE (43.6% vs 7.1%, respectively; *p* < 0.001) and lymph node metastasis (51.5% vs 7.1%, respectively; *p* < 0.001). Moreover, patients treated with 100 mCi, compared to those treated with 30 mCi, more frequently had microscopic ETE (52.4% vs. 11.9%, respectively; *p* < 0.001) and lymph node metastases (63.2% vs. 11.9%, respectively; *p* < 0.001).

**Table 2:** Clinicopathological features at diagnosis in 469 patients with thyroid cancer

Sex	
Female	350 (74.6%)
Male	119 (25.4%)
*Age*	46.1 (14.3)
*Papillary Histotype*	
No	15 (3.2%)
Yes	454 (96.8%)
*Aggressive histological variant*	
No	432 (92.1%)
Yes	37 (7.9%)
*Tumor Grade*	
Well differentiated	422 (90.0%)
Moderately differentiated	45 (9.6%)
Poorly differentiated	2 (0.4%)
*Margins of surgical resection*	
Negative	454 (96.8%)
Positive	15 (3.2%)
*Tumor size* (mm; mean, SD)	16.1 (10.6)
*Multifocality*	
No	329 (70.1%)
Yes	140 (29.9%)
*Bilaterality*	
No	367 (78.3%)
Yes	102 (21.7%)
*Vascular invasion*	
No	424 (90.4%)
Yes	45 (9.6%)
*M*icroscopic extra-thyroid extension	
No	316 (67.4%)
Yes	153 (32.6%)
*Lymph node metastases (N1a/b)*	
No	290 (61.8%)
Yes	179 (38.2%)

**Table 3:** Clinicopathological features and outcome in 469 patients with thyroid cancer according to radioactive iodine (RAI)

	Treated with RAI	Not treated with RAI	
	*N* = 328	*N* = 141	*P* value
*Sex*			0.33
Female	249 (75.9%)	101 (71.6%)	
Male	79 (24.1%)	40 (28.4%)	
*Age at diagnosis*	45.4 (14.7)	47.6 (13.1)	0.12
*Papillary Histotype*			0.77
No	11 (3.4%)	4 (2.8%)	
Yes	317 (96.6%)	137 (97.2%)	
*Aggressive histological variant*			0.008
No	295 (89.9%)	137 (97.2%)	
Yes	33 (10.1%)	4 (2.8%)	
*Tumor Grade*			0.023
Well differentiated	286 (87.2%)	136 (96.5%)	
Moderately differentiated	40 (12.2%)	5 (3.5%)	
Poorly differentiated	2 (0.6%)	0 (0.0%)	
*Surgical margins of resection*			0.78
Negative	318 (97.0%)	136 (96.5%)	
Positive	10 (3.0%)	5 (3.5%)	
*Tumor size* (mm; mean, SD)	16.6 (12.0)	14.9 (6.2)	0.13
*Multifocality*			<0.001
No	210 (64.0%)	119 (84.4%)	
Yes	118 (36.0%)	22 (15.6%)	
*Bilaterality*			<0.001
No	240 (73.2%)	127 (90.1%)	
Yes	88 (26.8%)	14 (9.9%)	
*Vascular invasion*			0.004
No	288 (87.8%)	136 (96.5%)	
Yes	40 (12.2%)	5 (3.5%)	
*Microscopic extra-thyroid extension*			<0.001
No	185 (56.4%)	131 (92.9%)	
Yes	143 (43.6%)	10 (7.1%)	
*Lymph node metastases*			<0.001
No	159 (48.5%)	131 (92.9%)	
Yes	169 (51.5%)	10 (7.1%)	
*Biochemical/structural recurrence*			<0.001
No	287 (87.5%)	138 (97.9%)	
Yes	41 (12.5%)	3 (2.1%)	
*Disease status at last visit*			0.014
Not evidence of disease	265 (80.8%)	129 (91.5%)	
Indeterminate response	37 (11.3%)	10 (7.1%)	
Biochemical incomplete response	12 (3.7%)	0 (0.0%)	
Structural incomplete response	14 (4.3%)	2 (1.4%)	
*Follow-up length* (months; median, 25–75 IQR)	64.9 (48.6–75.8)	64.2 (42.3–79.9)	0.74

### Identification of risk factors for PTC recurrence

Biochemical or structural recurrence during follow-up were more prevalent among patients in the RAI Group compared to those in the No RAI Group (Table 3). In detail, among patients in the RAI Group, 17 (41.5%) had BIR, and 24 (58.5%) had SIR, while all patients with recurrence in the No RAI Group had SIR. All SIR cases were in the neck lymph nodes and median time to recurrence was 56.8 months (25–75 IQR = 30.1–71.4) in RAI group, and 61.7 months (25–75 IQR = 34.7–78.4) in the No RAI group. Additional treatment (surgery and/or RAI) was necessary in 34 out of 41 patients in the RAI Group: 21 received only RAI, 3 underwent only surgery, and 10 required both RAI and surgery. In the No RAI Group, 1 out of 3 patients required additional treatment, and this patient was treated with RAI. To identify risk factors of tumor recurrence, we performed logistic regression analysis in these 469 patients (Table 4). Univariate logistic regression analysis revealed that the risk of recurrence was associated with male sex (OR = 2.74, 95%CI = 1.45–5.18; *p* = 0.002), tumor size (OR = 1.03, 95%CI = 1.01–1.06; *p* = 0.011), microscopic ETE (OR = 3.05, 95%CI = 1.62–5.74; *p* = 0.001) and lymph node metastases (OR = 4.46, 95%CI = 2.27–8.79; *p* < 0.001). By the other hand, age at diagnosis, papillary histotype, aggressive histologic variant, tumor grade, surgical resection margins, multifocality and bilaterality, vascular invasion were not associated with the risk of recurrence. Multivariate logistic regression analysis indicated that male sex (OR = 2.68, 95%CI = 1.31–5.50; p = 0.007), tumor size (OR = 1.03, 95%CI = 1.00–1.06; *p* = 0.017), microscopic ETE (OR = 2.53, 95%CI = 1.25–5.10; *p* = 0.010) and lymph node metastases (OR = 4.12, 95%CI = 1.88–9.04; *p* < 0.001) were independent predictors of recurrence. The tumor size threshold most strongly associated with recurrence was >15 mm. Taken together these data indicate that, in accordance with previous studies, histologic feature of tumor aggressiveness at diagnosis are predictive factors of tumor recurrence.

**Table 4:** Logistic regression analysis to evaluate factors associated with biochemical/structural recurrence

	Univariate analysis			Multivariate analysis		
	Odds ratio	95% CI	*P* value	Odds ratio	95% CI	*P* value
Male sex	2.74	1.45–5.18	0.002	2.68	1.31–5.50	0.007
Age at diagnosis	1.00	0.98–1.02	0.812			
RAI treatment	6.57	2.00–21.59	0.002	2.32	0.62–8.69	0.21
Papillary histotype	0.66	0.15–3.04	0.596			
Aggressive histological variant	1.57	0.58–4.27	0.373			
Tumor grade	1.93	0.88–4.21	0.099			
Surgical margins of resection	0.68	0.09–5.32	0.716			
Multifocality	1.39	0.72–2.65	0.323			
Tumor size	1.03	1.01–1.06	0.011	1.03	1.00–1.06	0.017
Bilaterality	0.66	0.28–1.52	0.327			
Vascular invasion	1.56	0.62–3.93	0.343			
Microscopic extra-thyroid extension	3.05	1.62–5.74	0.001	2.53	1.25–5.10	0.010
Lymph node metastases	4.46	2.27–8.79	0.000	4.12	1.88–9.04	<0.001

### Effect of radioiodine on patient risk stratification at last follow-up visit

At the last follow-up visit, in RAI group, 265 patients (80.8%) had an excellent response, 37 patients (11.3%) had IndR, 12 patients (3.7%) had BIR and SIR was identified in 14 patients (4.3%). In No RAI Group, 129 patients (91.5%) had an ER, 10 patients (7.1%) had IndR, 0 patients had BIR and SIR was identified in 2 patients (1.4%). Therefore, at the last follow-up biochemical/structural disease was identified in 26 patients (8.0%) of the RAI Group (median follow-up time since diagnosis= 64.9 months, 25–75 IQR = 48.6–75.8) and in 2 patients (1.4%) of the No RAI Group (median follow-up time since diagnosis= 64.2 months, 25–75 IQR = 42.3–79.9) (Table 3). Moreover, no significant difference in outcome was observed between patients treated with 30 mCi and those treated with 100 mCi (*p* = 0.13).

### Effect of radioiodine treatment on PTC recurrence

Since radioiodine treatment may exert a protective effect on tumor recurrence and clinicians’ preference may be in favor of RAI treatment for patients with more aggressive clinicopathological features at diagnosis, we aimed to estimate unbiased treatment effects. Hence, we analyzed our data by the inverse probability weighted regression adjustment after balancing the distribution of factors influencing treatment assignment and recurrence risk between RAI-treated and RAI-untreated patients. We observed that the potential outcome means (i.e., the estimated percentage of biochemical/structural recurrence) was 9.6% (95% CI = 6.3–12.9%) and 15.9% (95% CI = 11.1–20.7%) for patients who were treated or untreated with RAI, respectively (*p* = 0.021). Thus, if all patients were treated with RAI the estimated risk of biochemical/structural recurrence would have been reduced by 42% (OR = 0.58, 95% CI = 0.35–0.91; *p* = 0.018) than if no patient had received the RAI treatment. Based on the number of independent risk factors for recurrence identified through multivariate logistic regression analysis (male sex, tumor size >15 mm, presence of microscopic ETE and lymph node metastases, Table 4), we categorized our patients into four subgroups: those with no risk factors (*n* = 92; 60 of the No RAI and 32 of the RAI Group), one risk factor (*n*. = 134; 52 of the No RAI and 22 of the RAI Group), two risk factors (*n.*= 156; 25 of the No RAI and 131 of the RAI Group), and three or more risk factors (*n.*= 87; 4 of the No RAI and 83 of the RAI Group). Compared to patients with no risk factors, the recurrence risk was not significantly different in patients with one risk factor (OR = 5.02, 95% CI = 0.61–41.48, *p* = 0.14). However, the recurrence risk was statistically significantly higher in patients with two risk factors (OR = 8.97, 95% CI = 1.16–69.40, *p* = 0.036) and those with three or more risk factors (OR = 30.80, 95% CI = 4.05–234.31, *p* = 0.001). When adjusted for RAI therapy, the recurrence risk was not significantly different between patients with zero and patients with either one or two risk factors. Nevertheless, it remained significantly higher for those with three or more risk factors (OR = 18.09, 95% CI = 2.25–145.58, *p* = 0.007).

## Discussion

Patients with low to intermediate risk of recurrence represent a very heterogeneous subgroup of DTC, presenting with one or more pathological features of worse biological behavior and an estimated risk of recurrence up to 30% [9]. In this group of patients, risk classification is not solely based on a single feature but rather a combination of several factors. Although clinician’s decision mainly depends on the standard clinicopathologic features, a critical role is also played by additional parameters including the patient’s overall health and individual preferences, as well as local factors (i.e. the quality of preoperative and post-operative US evaluations, experience of the operating surgeon). Current evidences derive from retrospective studies and are conflicting to support the use of RAI for reducing the risk of recurrence and improving disease-specific survival. Therefore, guidelines suggest that RAI has no definitive proven benefit and leave to the clinician the choice of “consider” whether treating patients with low to intermediate risk or not. The INTERMEDIATE study is a multicentric phase III trial (NCT04290663) and is now enrolling patients with DTCs at intermediate risk with the aim of comparing two strategies: systematic RAI administration versus decision of radioiodine treatment guided by a post-operative work-up based on serum Tg values and diagnostic RAI scintigraphy (estimated study completion date February 2033). Only such as a prospective trial may warrant the body of evidence to definitively support the use of RAI for intermediate-risk patients. Because recurrences can sometimes manifest years later, sometimes exceeding five years [17, 18], clinical trials involving patients with thyroid cancer demand extensive follow-up durations, typically deemed time-intensive and entailing costly data quality monitoring processes. To obviate at this inconvenient, propensity score matching studies may be the most appropriate compromise. Results of propensity score matching studies are often in agreement with those obtained from randomized clinical trials examining the same clinical questions [19]. In our study we enrolled a number of patients similar to that estimated for the INTERMEDIATE trial (469 vs 476, respectively) and observed that patients treated with RAI had a higher risk of biochemical/structural recurrence than patients not treated (12.5% vs 2.1%, respectively). This finding was due to the presence of patients with more aggressive histological features at diagnosis that led clinicians to treat them with RAI (i.e. selection bias). Therefore, to minimize the effects of selection bias from non-randomized treatment assignment, we calculated the potential outcome mean and observed that RAI treatment significantly reduced the estimated risk of biochemical/structural recurrence by 42% (OR = 0.58; 95% CI = 0.35–0.91, *p* = 0.018). This finding highlights the real benefit from RAI treatment in this setting of patients. However, we cannot conclusively establish that RAI treatment is the optimal option for every patient with low to indeterminate risk given the significant variability in multifactorial parameters that assign different risk levels to each individual. Rather, our findings might lend support to the utilization of RAI treatment across the entire population of such risk category.

The identification of some risk factors may help clinicians to better identify which patients is suitable for RAI treatment. We found that biochemical/structural recurrence was more likely observed in either male patients or larger tumors or in the presence of microscopic ETE or lymph node metastases.

To predictive role of male sex is controversial. In a recent study, the authors revealed that male patients with PTC tended to exhibit certain unfavorable clinicopathological features more frequently than their female counterparts, including angioinvasion, lymph node metastases, and larger tumor size ( > 40 mm), which could potentially influence the response to initial therapy and the final disease status [20]. These findings were not confirmed by two independent studies where authors revealed that after controlling for selection bias using propensity score matching, male sex was no longer a risk factor for poor prognosis in patients with DTC treated with RAI [21, 22].

Tumor size is a well-recognized risk factor for recurrence in DTC patients. Tumors larger than 4 cm or larger than 2 cm in patients aged >55 may exhibit a higher risk of recurrence thus prompting closer post-operative monitoring and potentially more aggressive therapeutic and follow-up strategies [23, 24].

The prognostic significance of microscopic ETE in PTC is also debated [25]. Some studies have suggested that microscopic ETE may not significantly affect the risk of death or recurrence, while others have supported its inclusion in risk stratification models. In a recent longitudinal multicenter study authors observed that the combination of microscopic ETE and tumor size >2 cm may offer a more accurate prediction of disease persistence [26].

Lymph node metastasis is a well-recognized risk factor of DTC recurrence. Recently, Ruan et al. analyzed a cohort of 5241 papillary thyroid microcarcinoma and revealed that lymph node metastasis (LNM), especially lateral LNM, is a significant independent risk factor for tumor recurrence, with high HR of 3.03 for central LNM and 11.14 for lateral LNM [27].

Recently, has been demonstrated that non-excellent response at both one-year and final visit was observed in approximately half of the patients who had 3 or more intermediate risk factors, indicating that these individuals may benefit from increased monitoring and more intensive treatment [28]. Similarly, we observed that RAI treatment significantly reduced the risk of tumor recurrence in patients with low to intermediate recurrence risk. Our data showed that RAI did not affect recurrence risk in patients with only one risk factor, but a significant benefit was observed in those with two risk factors. According to the IPWRA analysis, we observed that RAI was associated with a lower risk of recurrence in the overall study population. Subsequently, we subdivided our patients according to the number of risk factors and found that this benefit persisted for those with two risk factors. In contrast, patients with three or more risk factors had a higher risk of recurrence despite the RAI treatment. The identified risk factors were independently associated with recurrence; therefore, no single factor was more strongly associated with recurrence than another. Instead, it was the cumulative presence of multiple risk factors that increased the risk.

While this study offers valuable insights into the potential benefits of RAI treatment in low to intermediate risk DTC, caution is warranted in interpreting our findings due to the inherent limitations of retrospective analyses. Additionally, as a single-center study, the generalizability of the findings to other populations or settings may be limited, and the influence of unmeasured confounders impacting both treatment assignment and recurrence rates cannot be entirely ruled out.

Taking into consideration the above-mentioned studies, the prognostic factors in papillary thyroid cancer, including tumor size, microscopic ETE, and lymph node metastasis, demonstrate varying significance and underscore the complexity of DTC. Radioiodine therapy in low to intermediate risk patients is often defined on a case-by-case basis, resulting in high treatment variability among thyroid cancer centers. In our study, RAI reduces the risk of recurrent disease in selected patients, however, there is a need for further comprehensive investigations to refine risk stratification models for more precise patient management and definitively answer the question of whether to consider RAI therapy for low to intermediate DTC or not.

## Data Availability

The datasets used and/or analyzed during the current study are available from the corresponding author on reasonable request.
